# Langerhans cells: Central players in the pathophysiology of atopic dermatitis

**DOI:** 10.1111/jdv.20291

**Published:** 2024-08-19

**Authors:** Yi Pan, Mathias Hochgerner, Małgorzata Anna Cichoń, Theresa Benezeder, Thomas Bieber, Peter Wolf

**Affiliations:** ^1^ Department of Dermatology and Allergy University Hospital of Bonn Bonn Germany; ^2^ Department of Dermatology and Venerology Medical University of Graz Graz Austria; ^3^ Greater Bay Area Institute of Precision Medicine (Guangzhou), School of Life Sciences, Fudan University Shanghai China; ^4^ Institute of Science and Technology Austria (IST Austria) Klosterneuburg Austria; ^5^ CK‐CARE, Medicine Campus Davos Switzerland; ^6^ Department of Dermatology University Hospital of Zürich Zürich Switzerland

## Abstract

Atopic dermatitis (AD) is the most common chronic inflammatory skin disease worldwide. AD is a highly complex disease with different subtypes. Many elements of AD pathophysiology have been described, but if/how they interact with each other or which mechanisms are important in which patients is still unclear. Langerhans cells (LCs) are antigen‐presenting cells (APCs) in the epidermis. Depending on the context, they can act either pro‐ or anti‐inflammatory. Many different studies have investigated LCs in the context of AD and found them to be connected to all major mechanisms of AD pathophysiology. As APCs, LCs recruit other immune cells and shape the immune response, especially adaptive immunity via polarization of T cells. As sentinel cells, LCs are primary sensors of the skin microbiome and are important for the decision of immunity versus tolerance. LCs are also involved with the integrity of the skin barrier by influencing tight junctions. Finally, LCs are important cells in the neuro‐immune crosstalk in the skin. In this review, we provide an overview about the many different roles of LCs in AD. Understanding LCs might bring us closer to a more complete understanding of this highly complex disease. Potentially, modulating LCs might offer new options for targeted therapies for AD patients.

## Graphical abstract

Langerhans cells (LCs) have unique properties to maintain cutaneous immunity. In atopic dermatitis (AD), LCs contribute to a variety of pathophysiological mechanisms underlying AD, such as immune response modulation, microbiome sensing, skin barrier integrity and neuro‐immune communication. Despite numerous insights into their dual pro‐ and anti‐inflammatory functions, much remains unknown. Understanding the central role of LCs in AD might enable innovative approaches to treat this complex disease.

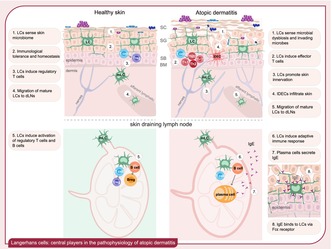

**Key points**

**Why was the study undertaken?**

This review aims to provide an overview about the current knowledge of the role of Langerhans cells in atopic dermatitis.

**What does this study add?**

Here, we review the contribution of LCs to the different pathophysiological mechanisms underlying AD, such as immune response modulation, microbiome sensing, skin barrier integrity and neuro‐immune communication, highlighting their dual pro‐ and anti‐inflammatory functions.

**What are the implications of this study for disease understanding and/or clinical care?**

Understanding the central role of LCs in AD might offer new ways to treat this very heterogeneous disease. We also point out, where our understanding is still lacking and further research is needed.

## INTRODUCTION

Langerhans cells (LCs) were discovered in 1868 and originally thought to be neurons,^1^ until they were recognized to be antigen‐presenting cells (APCs).^2^, ^3^, ^4^ For a long time, LCs were thought to be prototypical dendritic cells (DCs) and much of the early research into DCs was done with LCs. However, over time it became clear that LCs are a special type of cell.

**FIGURE 1:**
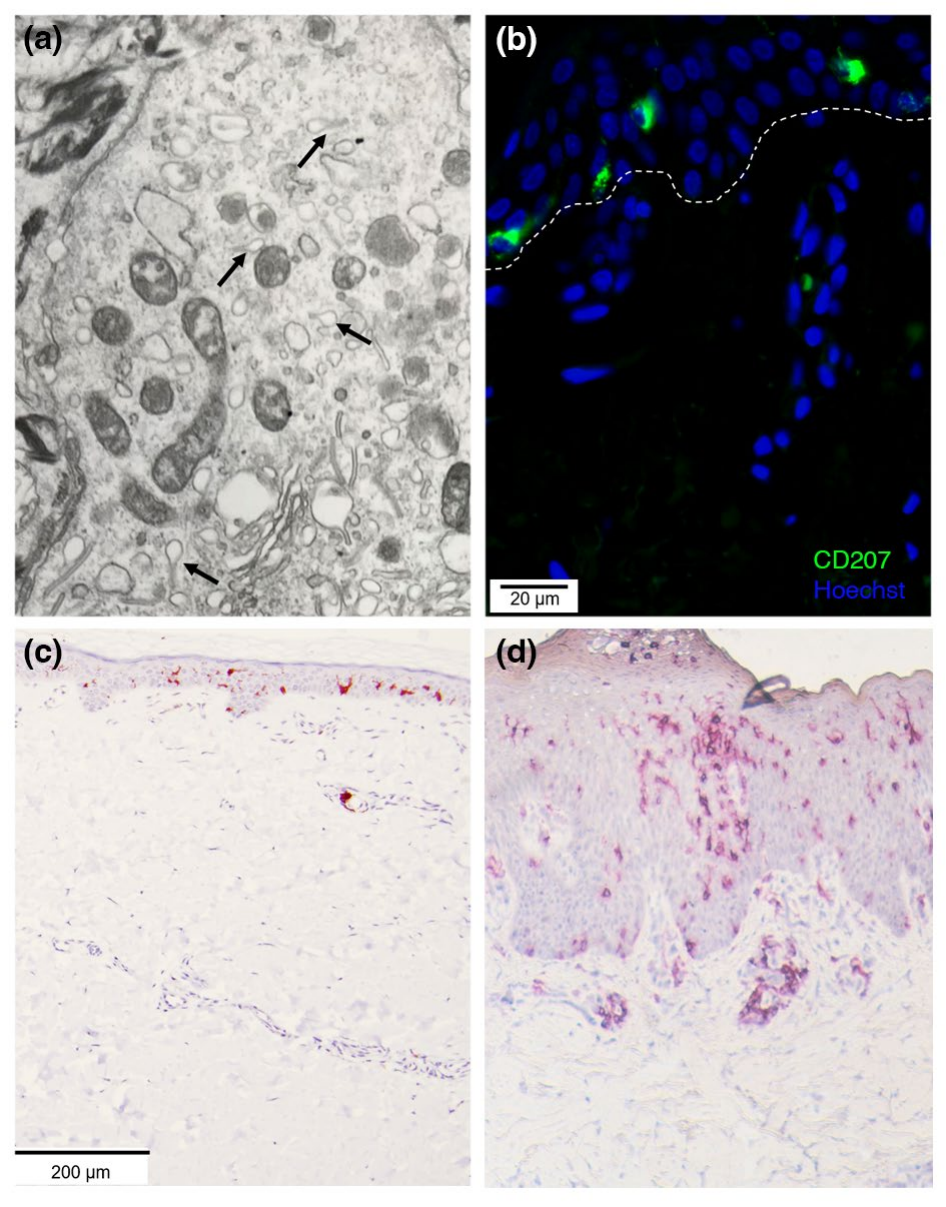
Human LCs in health and AD. (a) Electron microscopy image of cytoplasm of a Langerhans cell with arrows pointing at Birbeck granules. (b) Immunofluorescence image of healthy skin stained with anti‐CD207 (Langerin) antibody and Hoechst nucleic acid stain showing CD207+ Langerhans cells within the epidermis. The dotted line highlights the epidermal–dermal junction. (c, d) Representative immunohistochemistry images of healthy skin (c) and atopic dermatitis (d) stained with anti‐CD1a antibody. In healthy skin, epidermal CD1a + DCs are LCs. In AD skin, CD1a also stains infiltrating IDECs. AD, atopic dermatitis; LC, Langerhans cells; IDEC, inflammatory dendritic epidermal cells.

Langerhans cells were initially characterized ultrastructurally by their tennis racket‐shaped organelles composed of superimposed and zippered membranes, the so‐called Birbeck granules (BG; Figure 1a).^5^ These structures were later identified as expressing Langerin (CD207). Of note, ectopic expression of Langerin in fibroblasts leads to formation of BG in these cells.^6^ CD207 is helpful for identification of LCs by immunohistology or flow cytometry (Figure 1b). Human LCs are located in the basal and suprabasal layer of the epidermis and can extend their dendrites through tight junctions into the stratum corneum. This enables antigen recognition and capture both within the skin and on the skin surface.^7^ Recognition of antigen can result in LC activation and migration towards the nearest lymph node (LN). Considerable numbers of LCs have been found in skin draining LN but not in mesenteric LN.^8^

In healthy epidermis, LCs are the most abundant immune cells, comprising 2%–5% of all cells in the epidermis.^9^ Of note, females have more LCs than males.^10^ In both sexes, LC numbers decline with age.^11^ Langerin‐expressing DCs can also be found in the nasal, oral and cervical mucosa, in the foreskin, tonsils, tongue, the upper respiratory tract and the intestine.^12^, ^13^ However, mucosal LCs are of different origin and phenotype than epidermal LCs. Similarities and differences between mucosal and epidermal LCs were recently reviewed by Brand et al.^14^

Langerhans cells are radio‐resistant, therefore they are not depleted by irradiation in bone marrow chimera experiments. Otherwise, LCs are extremely sensitive. They can mature and emigrate upon contact with antigen, but LCs migrate also upon exposure to UV radiation, temperature‐, mechanical‐ and even psychological stress.^15^ In fact, the emigration of LCs upon UV exposure is so robust that in previously published studies ‘UV depletion’ of LCs was an accepted protocol. Of note, ‘UV depletion’ is not a depletion as we use the term today. Clean animal models of LC depletion use for example the Langerin‐DTR system. Therefore, one must be careful when reading selected literature about LCs and comparing their findings to newer data with modern experimental models.

The sensitivity of LCs to UV is of great interest in the field of phototherapy. While UV can ameliorate AD, the underlying mechanisms remain incompletely understood. In a mouse model of contact hypersensitivity, UV acts immunosuppressive and LCs are required for the generation of regulatory T cells (T_REG_).^16^ Results from a similar model showed that indeed epidermal LCs, not CD207^+^dDCs, are important for the anti‐inflammatory effects of UV treatment.^17^

## LC MARKERS

In‐vivo experiments are key in researching the immunopathology of diseases. The most common animal model for LC research is the mouse. However, although similar, there are differences between human and murine skin.^18^ Very basically, human epidermis and dermis are much thicker than murine, human skin is more firmly attached to underlying tissue and mice have a much higher hair density on the body than men.

In human skin, LCs comprise the vast majority of CD207‐expressing cells. Mouse skin contains a second population of CD207‐expressing APCs: CD207^+^ dermal DCs (dDCs). These cells, in contrast to epidermal LCs, are derived from bone marrow and not self‐renewing. They are CD103^+^XCR1^+^ and are excellent cross‐presenters. Thus, they can be seen as Type‐1 conventional dendritic cells. Markers for identifying human and mouse LCs and CD207^+^dDCs are shown in Table 1.

**TABLE 1:** LC markers in situ*.*

Markers	Synonyms	Human	Mouse	Mouse	Mouse
		Epidermal LCs	Epidermal LCs	Dermal cDC1	Dermal cDC2
CD1a		+	No mouse equivalent	No mouse equivalent	No mouse equivalent
CD1c		+	No mouse equivalent	No mouse equivalent	No mouse equivalent
CD11b		+/−	+	−	+
CD11c		+	+	+	+
CD24		−	+	+	−
C103		−	−	+	−
CD172a	SIRP‐alpha	+	+	−	+
CD205	DEC‐205	+	+	+	+
CD207	Langerin	+	+	+	−
CD209	DC‐SIGN	−	+	−	+
CD324	E‐Cadherin	+	+	+	+ (low?)
CD326	EpCAM	+	+	−	−
CLEC9a	DNGR‐1	−	−	+	−
F4/80		Not tested	+	−	+
MHC‐II		+	+	+	+
XRC1		−	−	+	−

Abbreviation: LCs, Langerhans cells.

Of note, in mice LCs and CD207^+^dDCs have different functions. Epidermal LCs seem to be more anti‐inflammatory/tolerogenic, while dermal CD207^+^dDCs seem to be more pro‐inflammatory. Different protocols for deleting LCs affect either one or both of these populations. The overlaps and differences between these two cell populations are the reason for much confusion in the literature.

### LC ontogeny

Epidermal LCs are an embryonically derived, self‐renewing, bone marrow‐independent cell population.^19^ Originally, they were classified as DCs, but they also display similarities with macrophages (Mac).^20^ Patients with GATA2 or IRF8 mutations show normal amounts of LCs. As these mutations affect DC numbers, LC development and maintenance are separate from plasmacytoid and conventional DCs (pDCs/cDCs).^21^ Lineage tracing studies in mice^19^, ^22^ have shown that LCs express both Zbtb46 and Mafb, giving them a dual identity of DCs and Mac.^23^

Langerhans cells share their origin with alveolar macrophages, microglia and Kupffer cells: Precursors from the embryonic yolk sac and the fetal liver seed the epidermis during embryogenesis. LC precursors can be observed in developing mouse skin as early as embryonic day 10.5.^19^ Between Days 2 and 7 after birth, these cells undergo a proliferative burst and form the LC network. LCs fully differentiate and upregulate MHC‐II, CD11c and finally CD207.^24^ The network settles to adult morphology around 3 weeks after birth.^25^

In the steady state, adult epidermal LCs have a half‐life of about 2 months. Even in homeostasis, LCs constantly mature and migrate to the draining LN in low amounts. The network is maintained by proliferation of LCs in situ. Normally, about 5% of LCs are proliferating at any given time.^24^ This self‐renewal is one of the defining features of LCs and has been demonstrated in multiple models. In parabiosis experiments, LCs remain of donor origin.^26^, ^27^, ^28^ In human allografts, LCs have been found to still be of donor origin 10 years after transplantation.^29^ This sets LCs apart from DCs, which have a higher turnover rate and arise from bone marrow precursors.^30^ Mucosal CD207^+^DCs (and in mouse CD207^+^dermal DCs) are also bone marrow‐derived and not self‐renewing.^14^

In case of mass emigration of LCs, like in acute inflammations, the LC network is re‐constituted from blood monocytes. These short‐lived monocyte‐derived LCs are replaced by long‐lived, non‐monocyte‐derived LCs over the course of 3 weeks. In case of a total depletion of LCs, the LC network can also be reconstituted from an as‐yet unidentified non‐monocyte bone marrow precursor in an ID2‐dependent manner.^31^

Langerhans cells depend in their development on the cytokine TGF‐β1.^32^, ^33^ However, active TGF‐β1 only appears in the epidermis after birth. Therefore, LC precursors seed the epidermis independently of TGF‐β1.^34^ The emergence of TGF‐β1 coincides with the proliferative burst and expression of CD207. While TGF‐β1 is absent, the prenatal and immediately postnatal epidermis does contain other proteins of the TGF‐β‐superfamily, most notably BMPs. In vitro experiments show that BMP7 can stimulate differentiation of LCs from human CD34^+^ cord blood stem cells, just like TGF‐β1. However, BMP7‐generated LCs show a different phenotype than TGF‐β1‐LCs. Most notably, BMP7‐LCs show active proliferation, altered expression of surface markers and no BG. In adult epidermis, both cytokines are present: The basal layer of the epidermis shows high levels of BMP7, while active TGF‐β1 is confined to the suprabasal layers. Proliferating LCs are only found in the BMP7‐dominated areas. Therefore, it is hypothesized that BMP7‐LCs represent the self‐renewing LCs, which then migrate upwards into the TGF‐β‐rich strata for terminal differentiation.^35^

### LC function

The primary function of LCs is the recognition and presentation of antigen. As professional APCs, they express an extensive repertoire of pattern recognition receptors (PRRs), mostly C‐type lectins (CLRs) and toll‐like receptors (TLRs). CLRs detect glycans, including those expressed by commensal and pathogenic bacteria. The main CLR expressed by LCs is Langerin (CD207). Other CLRs like mannose receptor (CD206) are only expressed under special conditions.^35^ CLR expression, glycan specificity and bacteria recognition have been thoroughly reviewed by Mnich et al.^36^

Toll‐like receptors are a large family of PRRs. TLRs 1, 2, 4, 5 and 6 detect extracellular molecular patterns such as lipopeptides, lipopolysaccharide, mannan, phospholipids, flagellin, zymosan and viral envelope proteins. TLRs 3 and 7–10 are expressed on endosomal membranes and recognize different forms of nucleic acids, for example dsRNA or CPG DNA.^37^ Human LCs have been described to express TLRs 1 through 10,^38^ but different publications report very different findings.^39^, ^40^, ^41^ A reason for these differences in the literature could be different types of LCs used (epidermal, mucosal or monocyte‐derived LCs), different methods of analysis (RNA‐Seq and flow cytometry) or different conditions (steady state and disease models). Interestingly, all studies comparing LCs to cDCs find significant differences in expressed TLRs. Flacher et al, combining qPCR with functional experiments, demonstrate that LCs functionally express TLRs 1, 2, 3, 5, 6 and 10, but not 4, 7 or 8. This specific expression pattern suggests that LCs might be specializing in defence against gram‐positive bacteria, but are less active against gram‐negative bacteria.^42^

As professional APCs, LCs can present antigen via MHC‐I and MHC‐II, as well as CD1a. Intracellular antigen is presented via MHC‐I to CD8^+^ T cells. Extracellular antigen is presented via MHC‐II to CD4^+^ T cells. LCs can also cross‐present, that is, present extracellular antigen to CD8^+^ T cells.^43^, ^44^

Upon contact with antigen, LCs may ‘mature’. In APCs, maturation means a series of phenotypical and functional alterations: they reduce their adhesion to the surrounding tissue and become highly mobile. They downregulate adhesion molecules, upregulate CCR7 and downregulate CCR6.^45^ This allows for their migration out of the tissue and to the next draining LN. LC maturation with the switch from epithelial to mesenchymal characteristics is often compared to the epithelial‐to‐mesenchymal transition (EMT) observed in metastasizing cancer cells.^46^

Mature LCs downregulate phagocytosis, but upregulate MHC‐II and the co‐stimulatory molecules CD40, CD80, CD86 and CD83. This allows for antigen presentation and immune activation.^47^ Activated, mature LCs can promote inflammation by secreting pro‐inflammatory cytokines, activating naïve T cells and re‐stimulating already primed T cells. LCs are able to polarize T cells towards Th1, Th2, Th17 or Th22 depending on the context.^48^, ^49^, ^50^

Langerhans cells are important for defence against pathogens. With their dendrites extending through tight junctions, LCs constantly survey the skin surface. Application of *Staphylococcus aureus* (*S. aureus*) exfoliating toxin (ET) to mouse skin did not compromise the epidermis and ET did not penetrate through tight junctions. Still, exposed mice developed neutralizing IgG against ET and were protected in subsequent intraperitoneal injections of ET. Mice depleted of LCs did not develop immunity after patch immunization. Therefore, LCs can establish adaptive immune defences against pathogens which have not even penetrated the body yet.^51^

Langerhans cells are especially important for the defence against viral infections. Their signature molecule CD207/Langerin can capture viral particles and internalize them for degradation in their BG.^52^ In several mouse models of viral infection, deletion of LCs leads to decreased cytotoxic T‐cell responses, higher viral loads and increased lethality.^53^, ^54^ Similarly, in Langerin‐DTR mice the immune response to leishmaniasis is impaired. However, Brewig et al.^55^ demonstrated that in this case the critical cells are CD207^+^dDCs.

Langerhans cells also have important anti‐inflammatory functions. In in‐vivo models of contact hypersensitivity, deletion of epidermal LCs exacerbates inflammation, while deletion of CD207^+^dDCs ameliorates inflammation.^56^ Here, epidermal LCs are mostly anti‐inflammatory/pro‐tolerogenic, while CD207^+^dDCs are pro‐inflammatory. Further research revealed that LCs can induce immunological tolerance to haptens by pushing allergen‐specific CD8^+^ T cells into anergy or deletion and expanding T_REGs_. Loss of LCs breaks this immunological tolerance and can result in inflammatory diseases.^57^, ^58^

Steady‐state LCs are highly phagocytic. Via the TAM‐kinase Axl, LCs silently clear apoptotic cells, preventing immune activation and autoimmunity.^59^ Deletion of TAM kinases results in a loss of epidermal LCs followed by spontaneous skin inflammation.^60^ In a mouse model of lupus, deletion of LCs leads to increased levels of auto‐antibodies and accelerated dermatitis.^61^ LCs are also important for maintaining tolerance towards skin commensals. In the steady state, LCs strongly favour induction of T_REGs_ over induction of anti‐bacterial effector T cells.^62^

Therefore, LCs can act both pro‐ and anti‐inflammatory, depending on the situation.

## ATOPIC DERMATITIS

Atopic dermatitis (AD) is the most common chronic inflammatory skin disease worldwide. Approximately 80% of cases begin in infancy or childhood, the rest develop in adulthood. The point prevalence in children varies from 2.7% to 20.1% in different countries and in adults from 2.1% to 4.9%.^63^

Risk factors for AD include exposure to lower temperatures, lower humidity, tobacco and air pollutants. These effects were more pronounced in children younger than 7 years and in women.^64^ These findings are of concern, especially in regard to rising urbanization and climate change.

Clinical manifestations of AD are sensitive and dry skin and localized or disseminated eczematous lesions, usually accompanied by severe pruritus.^65^ AD belongs to the spectrum of atopic diseases, which also includes food allergies, allergic asthma and allergic rhino‐conjunctivitis. All these atopic diseases may occur in different combinations.^63^, ^66^, ^67^ Patients with AD have a significantly increased risk of arthritis, Sjögren syndrome, Crohn's disease, vitiligo, alopecia areata, pernicious anaemia, ulcerative colitis, rheumatoid arthritis and hypothyroidism.^68^

Atopic dermatitis is a very heterogeneous disease and can be divided into different phenotypes and clinical manifestations defined by ethnicity, disease onset, disease severity, chronic versus acute, intrinsic versus extrinsic (IgE level), paediatric versus adult and inflammatory signature.^69^ This variety of endotypes makes it difficult to uncover a common pathophysiology and to develop therapies according to the one‐size‐fits‐all model.

Mild and moderate forms of AD are mainly treated topically, with emollients, pH‐adjusted moisturizers and anti‐inflammatory drugs like corticosteroids.^70^ Moderate and severe forms are treated systemically with biologics or JAK inhibitors aiming at the Th2‐immune response. Finally, AD can be treated successfully with phototherapy. Current and upcoming therapies have been reviewed in Bieber et al.^63^

### Pathophysiology of AD

The pathophysiology of AD is very complex and although many factors and processes are known, not all of these are present in all endotypes of AD. Genetic predisposition is a major risk factor for AD. It has been estimated that up to 90% of AD cases in Europe are inherited.^71^ Mutations associated with AD are related to the skin barrier and to immune regulation. Of note, although the clinical manifestations are similar, risk genes of AD differ substantially between cohorts from different ethnicities.^72^

Atopic dermatitis is a distinctly T‐cell‐driven disease with a strong IL‐13 signature.^73^ AD was originally viewed as a Th2‐driven disease, but recent data show a more complex picture. In European and American patients, Th2 and Th22 dominate the acute phase with Th1 following in the chronic phase of AD. Asian patients show higher levels of Th17 and Th22 responses, while African patients show almost no Th1 and Th17 and lower levels of Th22 involvement.^74^ Lesional AD skin also contains increased amounts of basophils, mast cells (MC), DCs, eosinophils and macrophages.^75^, ^76^, ^77^ Most cases of AD show high levels of specific IgE to foreign and self‐antigen, indicating an involvement of the humoral arm of the adaptive immune system.^78^ Indeed, systemic anti‐allergic therapy can ameliorate some forms of AD.^79^

Atopic dermatitis is furthermore associated with a compromised skin barrier. In AD patients, even non‐lesional skin shows increased trans‐epidermal water loss (TEWL).^80^ Mutations in filaggrin and claudin, key components of the skin barrier, are among the alleles with the strongest association with AD.^81^, ^82^

Whether it is a consequence or a cause of the compromised skin barrier, AD is associated with significant changes in the skin microbiome. Most prominently, AD skin displays an overgrowth of *S. aureus*.^83^ Not all patients with AD do have *S. aureus* colonization of their skin, but levels of *S. aureus* correlate with disease severity and AD patients without *S. aureus* colonization have less severe skin symptoms.^84^ Of note, just like the barrier disruption, the microbiome in AD patients is also changed in non‐lesional skin.^85^ Treatment with corticosteroids ameliorates skin lesions and leads to decreased microbial dysbiosis.^86^ On the other hand, normalizing the microbiome can lead to an amelioration of the disease.^87^, ^88^ Therefore, microbial dysbiosis is both a consequence of AD and a driver of the disease.

Neuro‐immune crosstalk is an important element of many diseases.^89^ Especially AD is associated with increased innervation of the skin.^90^ In mouse models, amelioration of AD correlates with reduced skin innervation.^91^ In AD lesions, the density of nerves in the epidermis is increased with nerves displaying altered morphology.^92^ Fitting to this observation, AD skin contains higher levels of nerve growth factor (NGF).^93^ In a mouse model of AD, blockade of NGF reduces the innervation of the skin and improves dermatitis and scratching behaviour.^94^

How all these factors connect and if all of them are important in all forms of AD is currently still unclear. A full understanding of the pathophysiology of AD will still require much research.

### LCs in AD

The prominence of LCs in the skin, as well as their dual function as potentially strong pro‐ and anti‐inflammatory cells, makes them cells of great interest in inflammatory skin diseases. Furthermore, genome‐wide association studies have found mutations in the gene encoding for CD207 to be strongly associated with risk of AD.^72^ LCs in AD show greater activation and maturation^95^ and increased proliferation^24^ (Figure 1c,d). In several different mouse models, deletion of LCs protects from AD.^96^ Xiao et al. elegantly demonstrated that specifically deletion of monocyte‐derived LCs, but not CD207^+^dDCs, ameliorates AD.^97^

It is assumed that LCs are actively involved in the pathophysiology of AD. They may however also be involved in anti‐inflammatory pathways ameliorating the disease. As of yet, the definitive role of LCs in AD remains unclear. However, LCs are involved in many key processes of AD, giving them a central role in this disease.

### LCs and immunology in AD

T cells are key players in the pathophysiology of AD, as exemplified by current treatments blocking T‐cell‐derived cytokines. Th2‐polarized helper T cells secrete the cytokines IL‐4 and IL‐13. Blockade of these cytokines or their receptors, for example with dupilumab, is a highly efficient treatment for AD.^98^ While T cells are strong effector cells, their activation, recruitment and polarization depend on APCs. LCs are the most common APCs in the skin and have been shown to be able to polarize T cells towards Th2, making them the obvious drivers of the Th2‐shift in AD.

Lesional skin of patients with AD contains high levels of the cytokine thymic stromal lymphopoietin (TSLP), produced by keratinocytes. In mice, overexpression of TSLP leads to an AD‐like disease. Interestingly, depletion of LCs abolishes AD in this model. Therefore, TSLP induces AD via LCs. Specifically, TSLP‐primed LCs recruit T cells to the skin via secretion of chemokines like CCL17 and CCL22 and polarize them towards a Th2‐phenotype.^96^, ^99^, ^100^

The best studied mode of activation for T cells is via MHC‐I and MHC‐II, presenting peptide antigen. However, T cells can also be activated by lipid antigen presented by CD1a. In AD, CD1a‐reactive T cells are strongly increased.^101^ CD1a is highly expressed on LCs.^102^ Therefore, LCs may drive AD pathogenesis via CD1a‐dependent activation of T cells.

One important downstream effect of Th2‐polarization is the induction of B‐cell differentiation towards IgE‐producing plasma cells. In mouse models, deletion of LCs leads to reduced levels of IgE, both in a model of OVA sensitization and in the steady state.^103^ AD has long been associated with high levels of IgE.^104^, ^105^ LCs bind IgE via different structures such as the low‐affinity receptor FcɛRII/CD23 and the high‐affinity receptor FcɛRI. Both receptors are expressed on LCs in AD.^106^, ^107^ FcɛRI expression on CD1a^+^ cells correlates with serum IgE levels in patients.^108^ Activation of IgE‐bound FcɛRI on LCs leads to secretion of chemokines such as IL‐16, which recruit more helper T cells, precursors of inflammatory dendritic epidermal cells (IDECs) and eosinophils.^109^ IDECs bind IgE via FcɛRI, but FcɛRI‐activated IDECs polarize T cells in a different direction, namely towards Th1.^110^

Therefore, there exists a feedback loop in the pathogenesis of AD: LCs initiate a Th2‐response, which leads to IgE‐production, which is in turn bound by the LCs. Their activation upon IgE ligation leads to the recruitment of further immune cells. IgE on IDECs, as a spin off of this loop, polarizes naïve T cells towards Th1, explaining the switch form Th2 in acute to Th1 in chronic AD lesions.

Another important cell type infiltrating the skin in AD are eosinophils.^111^ In‐vivo, disruption of the skin barrier and immune activation with peptidoglycan cause eosinophil infiltration in the skin. This is dependent on secretion of the chemokine CCL5 by LCs.^112^ In another mouse model of dermatitis, Langerin‐DTR depletion of LCs leads to reduced levels of eosinophils.^113^

Therefore, LCs play an important part in recruiting and polarizing immune cells, driving the pathogenesis of AD.

### LCs and skin barrier in AD

Atopic dermatitis skin is characterized by a genetically and inflammation‐driven disrupted skin barrier. AD skin also contains higher numbers of more active LCs. In several different mouse models, disruption of skin barrier led to increased numbers of LCs.^114^ Yoshida et al. suggested that due to their ability to penetrate tight junctions, LC may contribute to formation of skin lesions by having access to environmental allergens.^115^, ^116^

Mice with a deletion of EGFR in the epidermis (EGFR^Δep^) develop a phenotype resembling severe AD. This phenotype is rescued when the mice are kept under germ‐free conditions. Therefore, the skin inflammation in EGFR^Δep^mice is caused by invading microbes. Importantly, even germ‐free, non‐inflamed mice have a compromised skin barrier. In this model, skin barrier integrity loss is the initiating event of the disease, allowing for infection of bacteria into the skin and leading to the persistent inflammation.^117^ In EGFR^Δep^mice, the disease starts when hair follicles erupt through the skin. Normal closure of the skin barrier after this disruption is impaired in these mice.

In AD patients, numbers of LCs with dendrites reaching into the stratum corneum are increased fivefold.^115^ In normal skin, tight junctions close around LC dendrites to maintain the skin barrier.^7^ However, AD is very prominently associated with mutations in genes coding for skin barrier components. Therefore, an increased penetration of the tight junctions by LC‐dendrites, coupled with a defect in closing tight junctions, may be a contributing factor in the pathology of AD. A compromised skin barrier might attract more LCs, which might further compromise the barrier, leading to a vicious cycle driving the disease.

However, data from Lee et al. suggest an opposite hypothesis: in both Langerin‐DTA and Langerin‐DTR mice, two genetic models for deletion of LCs, the skin barrier is disturbed. Even in the steady state, the transepidermal water loss (TEWL) in these mice is higher than in WT controls. Also, mice without LCs show impaired skin barrier closure after tape stripping.^118^ According to these results, LCs contribute to the maintenance and repair of the skin barrier. The increased amount of LC‐dendrites in the tight junction area may be an attempt to ‘plug the holes’ in a compromised skin barrier.^119^

An intact skin barrier is essential for the tolerogenic function of LCs. Luo et al. injected OVA into the subcutis of mice, eliciting an anti‐OVA immune response. If OVA was applied topically to intact skin before the injection, the anti‐OVA response was attenuated significantly. This effect was lost in Langerin‐DTA mice. Therefore, topical application of an antigen can lead to immunological tolerance in an LC‐dependent manner. Cutaneous application of OVA on tape‐stripped skin also failed to elicit immunological tolerance.^120^ Therefore, the critical point of cutaneous tolerance is antigen sensing by immature LCs through an intact skin barrier. The disturbed skin barrier in AD may deprive the patient of an important immunoregulatory mechanism. This may continue to drive the disease or contribute to the emergence of allergic comorbidities.

Uncovering the exact connections between LCs and the skin barrier in human AD will still need further research, but modifying LCs might present a way to restore the skin barrier and ameliorate the disease.

### LCs and microbiome in AD

Microbial dysbiosis is very important in AD.^121^ The skin and its microbiome constantly interact with each other via cell–cell interactions, structural components or secreted bioactive molecules.^122^ This interaction goes both ways: Comparing germ‐free to SPF mice revealed over 2800 differentially regulated genes in the skin, demonstrating the enormous effect the microbiome has on the skin even in steady state.^123^ Conversely, changes in the immune system lead to alterations of the microbiome, even in the steady state.^124^ In healthy mice, depletion of LCs does not affect the composition of the skin microbiome.^125^ Germ‐free mice, compared to SPF mice, display reduced numbers of LCs in the epidermis.^126^ Therefore, LCs may be sensors of the microbiome, but not directly influence it.

In healthy skin, LCs induce T_REGs_. When activated, however, LCs expand effector and memory T cells and decrease T_REGs_.^127^ Metabolites from skin commensals can act on LCs and induce an anti‐inflammatory programme with secretion of IL‐10 and IDO. Such LCs can further suppress helper T‐cell proliferation and induce IL‐10 secretion in T cells.^128^

In AD, the skin is often colonized by an overabundance of *S. aureus*, while other species like *Staphylococcus epidermidis* (*S. epidermidis*) are reduced. As yet unpublished data from our group show that LCs primed with *S. epidermidis* lead to IL‐10‐secreting T cells, while LCs primed with *S. aureus* lead to proliferating T cells secreting pro‐inflammatory cytokines. Pathogens like *S. aureus* can drive T‐cell‐mediated inflammation via LCs. At the same time, LC‐translated anti‐inflammatory functions of skin commensals like *S. epidermidis* are lacking.

Iwamoto et al report that in AD LCs display significant downregulation of TLR2 and are therefore unresponsive to TLR2 ligands. Pam3Cys‐stimulated LCs from AD patients produce less IL‐6 and IL‐10, but more IL‐18.^129^ Therefore, in AD LCs react to the microbiome in an atypical way.

Not only the bacterial, but also the fungal skin microbiome is dysregulated in AD. Especially species of *Malassezia* have been associated with more severe AD.^75^ Of note, skin‐resident fungi are strong inducers of IgE responses.^130^ FcɛR1‐activated LCs secrete chemokines attracting T cells and IDEC precursors.^110^ This may contribute to the perpetuation of AD. The pathogenicity of fungi depends on the pH of the surroundings. AD skin is less acidic than healthy skin. This leads to increased release of allergens from skin‐resident fungi,^131^ leading to more IgE production. Changes in the fungal microbiome could very well be the basis of the AD‐specific expression of IgE receptors in LCs.

Taken together, both the presence of pro‐inflammatory microbes, as well as the absence of commensals which would induce tolerogenic programmes in LCs, are important factors in AD. Transplantation of healthy microbiome can ameliorate AD in mice.^88^ Clinical studies with microbiome transplantation in AD patients are already under way.^132^

### LCs and neuro‐immunology in AD

Skin inflammation often correlates with psychosomatic disorders. Psychological stress often leads to worsening of skin diseases.^133^, ^134^, ^135^, ^136^, ^137^ Chronic skin conditions in turn often cause stress, via self‐perceived unattractiveness of the patient or via the sensation of pain and itch. This vicious cycle contributes to persistence and worsening of chronic skin diseases.

In mice, deletion of LCs can reduce the number of specific nerve fibres.^138^, ^139^ In humans, demyelinating diseases or spinal cord injuries lead to loss of skin innervation, which in turn reduces numbers of LCs.^140^, ^141^ Thus, nerves and LCs in the skin are dependent on each other.

Zhang et al. demonstrated that long‐term loss of LCs leads to reduction of specific nerve fibres. This in turn leads to increased MC degranulation, which aggravates in‐vivo models of skin inflammation. However, depletion of skin nerves did not aggravate experimental AD. In other models, the opposite effect occurs: depletion of skin nerves ameliorates inflammation.^142^ Neuro‐immunology of the skin is very complex and will require more research to fully understand.

In AD patients, innervation of the epidermis is increased. This has been connected especially with the sensations of itch and pain.^143^ Depletion of these nerves ameliorates AD.^94^

In both animal models and patients, AD is associated with a dysregulation of neuropeptides. Specifically, in AD the levels of substance P (SP) are increased and the levels of calcitonin gene‐related peptide (CGRP) are decreased.^144^, ^145^ CGRP acts on LCs and impairs their ability to present antigen.^146^ Injection of CGRP downregulates inflammation in‐vivo.^147^ SP also acts on LCs,^148^ but acts pro‐inflammatory by enhancing LC activation, migration and antigen presentation, leading to enhanced T‐cell reactions and increased productions of immunoglobulins.^149^ In AD, SP levels correlate with NGF levels and with disease severity.^150^

Thus, inflammatory LCs may increase the innervation of the skin. Inflammatory nerves in turn may polarize LCs towards a pro‐inflammatory phenotype, thus creating an inflammation‐driving feedback loop.

## CONCLUSIONS

LCs play an important part in skin immunity. In the steady state, they maintain tissue homeostasis and immunological tolerance. At the same time, they provide immunity against invading and skin surface microbes. LCs are connected to all major events of AD pathophysiology. However, their net effect in this disease is still unclear. LCs might be drivers of AD or they might attempt to counteract the disease. Moreover, LCs may play different roles in different subtypes and stages of AD. The literature on this topic is challenging to understand; many different models have been used and the role of LCs may vary with the exact model. Different groups use epidermal LCs, CD207^+^dDCs or monocyte‐derived LCs. Moreover, in vitro experiments do not replicate the microenvironment of the skin and the microbiome, which are critical for LCs. Therefore, in vitro data may not reflect the in‐vivo situation very well.

More work is needed in order to deepen the understanding of LCs in the pathophysiology of AD. Studies about LC function in patients with different subtypes and stages of AD would be key to better understand their putative role in distinct subforms of AD. Hence, the behaviour of LCs in AD patients of different ethnicities and different dominant cytokine axes would be interesting to explore. Besides all challenges of investigation, LCs, as key players in the regulatory networks in AD, offer the opportunity to develop new treatment strategies and options, for example, by developing drugs modifying their function and shift them away from pro‐inflammatory action.

## AUTHOR CONTRIBUTIONS

YP: Conceptualization, funding acquisition, roles/writing—original draft and writing—review and editing. MH: Conceptualization, roles/writing—original draft and writing—review and editing. MAC: Conceptualization, visualization, roles/writing—original draft and writing—review and editing. TBe: Visualization. TBi: Supervision and writing—review and editing. PW: Funding acquisition, supervision and writing—review and editing.

## CONFLICT OF INTEREST STATEMENT

The authors declare no potential conflict of interest.

## Data Availability

Data sharing not applicable to this article as no data sets were generated or analysed during the review.

## References

[1] Langerhans P . Ueber die Nerven der menschlichen Haut. Archiv f Pathol Anat. 1868;44:325–337.

[2] Stingl G , Wolff‐Schreiner EC , Pichler WJ , Gschnait F , Knapp W , Wolff K . Epidermal Langerhans cells bear fc and C3 receptors. Nature. 1977;268(5617):245–246. 887158 10.1038/268245a0

[3] Schuler G , Steinman R . Murine epidermal Langerhans cells mature into potent immunostimulatory dendritic cells in vitro. J Exp Med. 1985;161:526–546. 3871837 10.1084/jem.161.3.526 PMC2187584

[4] Steinman RM , Cohn ZA . Identification of a novel cell type in peripheral lymphoid organs of mice. J Exp Med. 1973;137:1142–1162. 4573839 10.1084/jem.137.5.1142 PMC2139237

[5] Birbeck MS , Breathnach AS , Everall JD . An electron microscope study of basal melanocytes and high‐level clear cells (Langerhans cells) in vitiligo. J Invest Dermatol. 1961;37(1):51–64.

[6] Valladeau J , Ravel O , Dezutter‐Dambuyant C , Moore K , Kleijmeer M , Liu Y , et al. Langerin, a novel C‐type lectin specific to Langerhans cells, is an endocytic receptor that induces the formation of Birbeck granules. Immunity. 2000;12:71–81. 10661407 10.1016/s1074-7613(00)80160-0

[7] Kubo A , Nagao K , Yokouchi M , Sasaki H , Amagai M . External antigen uptake by Langerhans cells with reorganization of epidermal tight junction barriers. J Exp Med. 2009;206(13):2937–2946. 19995951 10.1084/jem.20091527 PMC2806471

[8] Stoitzner P , Holzmann S , McLellan AD , Ivarsson L , Stossel H , Kapp M , et al. Visualization and characterization of migratory Langerhans cells in murine skin and lymph nodes by antibodies against Langerin/CD207. J Invest Dermatol. 2003;120(2):266–274. 12542532 10.1046/j.1523-1747.2003.12042.x

[9] Romani N , Clausen BE , Stoitzner P . Langerhans cells and more: langerin‐expressing dendritic cell subsets in the skin. Immunol Rev. 2010;234(1):120–141. 20193016 10.1111/j.0105-2896.2009.00886.x PMC2907488

[10] Koyama Y , Nagao S , Ohashi K , Takahashi H , Marunouchi T . Sex differences in the densities of epidermal Langerhans cells of the mouse. J Invest Dermatol. 1987;88(5):541–544. 3572027 10.1111/1523-1747.ep12470104

[11] Thiers BH , Maize JC , Spicer SS , Cantor AB . The effect of aging and chronic sun exposure on human Langerhans cell populations. J Invest Dermatol. 1984;82(3):223–226. 6199432 10.1111/1523-1747.ep12260055

[12] Allam P , Niederhagen B , Bücheler M , Appel T , Betten H , Bieber T , et al. Comparative analysis of nasal and oral mucosa dendritic cells. Allergy. 2006;61:166–172. 16409191 10.1111/j.1398-9995.2005.00965.x

[13] Iijima N , Thompson JM , Iwasaki A . Dendritic cells and macrophages in the genitourinary tract. Mucosal Immunol. 2008;1(6):451–459. 19079212 10.1038/mi.2008.57 PMC2684461

[14] Brand A , Hovav AH , Clausen BE . Langerhans cells in the skin and oral mucosa: brothers in arms? Eur J Immunol. 2023;53:e202149499. 10.1002/eji.202149499 36811456

[15] Nakano Y . Stress‐induced modulation of skin immune function: two types of antigen‐presenting cells in the epidermis are differentially regulated by chronic stress. Br J Dermatol. 2004;151(1):50–64. 15270872 10.1111/j.1365-2133.2004.05980.x

[16] Schwarz A , Noordegraaf M , Maeda A , Torii K , Clausen BE , Schwarz T . Langerhans cells are required for UVR‐induced immunosuppression. J Invest Dermatol. 2010;130(5):1419–1427. 20090769 10.1038/jid.2009.429

[17] Taguchi K , Fukunaga A , Ogura K , Nishigori C . The role of epidermal Langerhans cells in NB‐UVB‐induced immunosuppression. Kobe J Med Sci. 2013;59(1):E1–E9. 23756657

[18] Zomer HD , Trentin AG . Skin wound healing in humans and mice: challenges in translational research. J Dermatol Sci. 2018;90(1):3–12. 29289417 10.1016/j.jdermsci.2017.12.009

[19] Hoeffel G , Wang Y , Greter M , See P , Teo P , Malleret B , et al. Adult Langerhans cells derive predominantly from embryonic fetal liver monocytes with a minor contribution of yolk sac‐derived macrophages. J Exp Med. 2012;209(6):1167–1181. 22565823 10.1084/jem.20120340 PMC3371735

[20] Guilliams M , Ginhoux F , Jakubzick C , Naik SH , Onai N , Schraml BU , et al. Dendritic cells, monocytes and macrophages: a unified nomenclature based on ontogeny. Nat Rev Immunol. 2014;14(8):571–578. 25033907 10.1038/nri3712 PMC4638219

[21] Collin M , Bigley V , Haniffa M , Hambleton S . Human dendritic cell deficiency: the missing ID? Nat Rev Immunol. 2011;11(9):575–583. 21852794 10.1038/nri3046

[22] Anderson DA , Dutertre CA , Ginhoux F , Murphy KM . Genetic models of human and mouse dendritic cell development and function. Nat Rev Immunol. 2021;21(2):101–115. 32908299 10.1038/s41577-020-00413-x PMC10955724

[23] Wu X , Briseno CG , Durai V , Albring JC , Haldar M , Bagadia P , et al. Mafb lineage tracing to distinguish macrophages from other immune lineages reveals dual identity of Langerhans cells. J Exp Med. 2016;213(12):2553–2565. 27810926 10.1084/jem.20160600 PMC5110021

[24] Chorro L , Sarde A , Li M , Woollard KJ , Chambon P , Malissen B , et al. Langerhans cell (LC) proliferation mediates neonatal development, homeostasis, and inflammation‐associated expansion of the epidermal LC network. J Exp Med. 2009;206(13):3089–3100. 19995948 10.1084/jem.20091586 PMC2806478

[25] Tripp CH , Chang‐Rodriguez S , Stoitzner P , Holzmann S , Stossel H , Douillard P , et al. Ontogeny of Langerin/CD207 expression in the epidermis of mice. J Invest Dermatol. 2004;122(3):670–672. 15086552 10.1111/j.0022-202X.2004.22337.x

[26] Ginhoux F , Greter M , Leboeuf M , Nandi S , See P , Gokhan S , et al. Fate mapping analysis reveals that adult microglia derive from primitive macrophages. Science. 2010;330(6005):841–845. 20966214 10.1126/science.1194637 PMC3719181

[27] Kanitakis J , Morelon E , Petruzzo P , Badet L , Dubernard JM . Self‐renewal capacity of human epidermal Langerhans cells: observations made on a composite tissue allograft. Exp Dermatol. 2011;20(2):145–146. 20707812 10.1111/j.1600-0625.2010.01146.x

[28] Merad M , Ginhoux F , Collin M . Origin, homeostasis and function of Langerhans cells and other langerin‐expressing dendritic cells. Nat Rev Immunol. 2008;8(12):935–947. 19029989 10.1038/nri2455

[29] Kanitakis J , Petruzzo P , Dubernard JM . Turnover of epidermal Langerhans' cells. N Engl J Med. 2004;351:2661–2662. 15602033 10.1056/NEJM200412163512523

[30] Deckers J , Hammad H , Hoste E . Langerhans cells: sensing the environment in health and disease. Front Immunol. 2018;9:93. 29449841 10.3389/fimmu.2018.00093 PMC5799717

[31] Sere K , Baek JH , Ober‐Blobaum J , Muller‐Newen G , Tacke F , Yokota Y , et al. Two distinct types of Langerhans cells populate the skin during steady state and inflammation. Immunity. 2012;37(5):905–916. 23159228 10.1016/j.immuni.2012.07.019

[32] Borkowski TA , Letterio JJ , Farr AG , Udey MC . A role for endogenous transforming growth factor beta 1 in Langerhans cell biology: the skin of transforming growth factor beta 1 null mice is devoid of epidermal Langerhans cells. J Exp Med. 1996;184(6):2417–2422. 8976197 10.1084/jem.184.6.2417 PMC2196398

[33] Strobl H , Riedl E , Scheinecker C , Bello‐Fernandez C , Pickl WF , Rappersberger K , et al. TGF‐beta 1 promotes in vitro development of dendritic cells from CD34+ hemopoietic progenitors. J Immunol. 1996;157(4):1499–1507. 8759731

[34] Yasmin N , Bauer T , Modak M , Wagner K , Schuster C , Koffel R , et al. Identification of bone morphogenetic protein 7 (BMP7) as an instructive factor for human epidermal Langerhans cell differentiation. J Exp Med. 2013;210(12):2597–2610. 24190429 10.1084/jem.20130275 PMC3832935

[35] Borek I , Koffel R , Feichtinger J , Spies M , Glitzner‐Zeis E , Hochgerner M , et al. BMP7 aberrantly induced in the psoriatic epidermis instructs inflammation‐associated Langerhans cells. J Allergy Clin Immunol. 2020;145(4):1194–1207.e11. 31870764 10.1016/j.jaci.2019.12.011

[36] Mnich ME , van Dalen R , van Sorge NM . C‐type lectin receptors in host defense against bacterial pathogens. Front Cell Infect Microbiol. 2020;10:309. 32733813 10.3389/fcimb.2020.00309 PMC7358460

[37] Wicherska‐Pawlowska K , Wrobel T , Rybka J . Toll‐like receptors (TLRs), NOD‐like receptors (NLRs), and RIG‐I‐like receptors (RLRs) in innate immunity. TLRs, NLRs, and RLRs ligands as immunotherapeutic agents for hematopoietic diseases. Int J Mol Sci. 2021;22(24). 10.3390/ijms222413397 PMC8704656 34948194

[38] Renn CN , Sanchez DJ , Ochoa MT , Legaspi AJ , Oh CK , Liu PT , et al. TLR activation of Langerhans cell‐like dendritic cells triggers an antiviral immune response. J Immunol. 2006;177(1):298–305. 16785525 10.4049/jimmunol.177.1.298

[39] Rozis G , Benlahrech A , Duraisingham S , Gotch F , Patterson S . Human Langerhans' cells and dermal‐type dendritic cells generated from CD34 stem cells express different toll‐like receptors and secrete different cytokines in response to toll‐like receptor ligands. Immunology. 2008;124(3):329–338. 18194273 10.1111/j.1365-2567.2007.02770.x PMC2440827

[40] van der Aar AM , Sylva‐Steenland RM , Bos JD , Kapsenberg ML , de Jong EC , Teunissen MB . Loss of TLR2, TLR4, and TLR5 on Langerhans cells abolishes bacterial recognition. J Immunol. 2007;178(4):1986–1990. 17277101 10.4049/jimmunol.178.4.1986

[41] Tajpara P , Schuster C , Schon E , Kienzl P , Vierhapper M , Mildner M , et al. Epicutaneous administration of the pattern recognition receptor agonist polyinosinic‐polycytidylic acid activates the MDA5/MAVS pathway in Langerhans cells. FASEB J. 2018;32(8):4132–4144. 29509510 10.1096/fj.201701090R PMC6053315

[42] Flacher V , Bouschbacher M , Verronèse E , Massacrier C , Sisirak V , Berthier‐Vergnes O , et al. Human Langerhans cells express a specific TLR profile and differentially respond to viruses and gram‐positive bacteria. J Immunol. 2006;177(11):7959–7967. 17114468 10.4049/jimmunol.177.11.7959

[43] Clausen BE , Stoitzner P . Functional specialization of skin dendritic cell subsets in regulating T cell responses. Front Immunol. 2015;6:534. 26557117 10.3389/fimmu.2015.00534 PMC4617171

[44] Fehres CM , Duinkerken S , Bruijns SC , Kalay H , van Vliet SJ , Ambrosini M , et al. Langerin‐mediated internalization of a modified peptide routes antigens to early endosomes and enhances cross‐presentation by human Langerhans cells. Cell Mol Immunol. 2017;14(4):360–370. 26456691 10.1038/cmi.2015.87 PMC5380941

[45] Barbaroux JB , Kwan WH , Allam JP , Novak N , Bieber T , Fridman WH , et al. Tumor necrosis factor‐alpha‐ and IL‐4‐independent development of Langerhans cell‐like dendritic cells from M‐CSF‐conditioned precursors. J Invest Dermatol. 2006;126(1):114–120. 16417226 10.1038/sj.jid.5700023

[46] Konradi S , Yasmin N , Haslwanter D , Weber M , Gesslbauer B , Sixt M , et al. Langerhans cell maturation is accompanied by induction of N‐cadherin and the transcriptional regulators of epithelial‐mesenchymal transition ZEB1/2. Eur J Immunol. 2014;44(2):553–560. 24165969 10.1002/eji.201343681

[47] Von Bubnoff D , Scheler M , Wilms H , Fimmers R , Bieber T . Identification of IDO‐positive and IDO‐negative human dendritic cells after activation by various proinflammatory stimuli. J Immunol. 2011;186(12):6701–6709. 21543643 10.4049/jimmunol.1003151

[48] Fujita H , Nograles KE , Kikuchi T , Gonzalez J , Carucci JA , Krueger JG . Human Langerhans cells induce distinct IL‐22‐producing CD4+ T cells lacking IL‐17 production. Proc Natl Acad Sci USA. 2009;106(51):21795–21800. 19996179 10.1073/pnas.0911472106 PMC2799849

[49] Furio L , Briotet I , Journeaux A , Billard H , Peguet‐Navarro J . Human Langerhans cells are more efficient than CD14(−)CD1c(+) dermal dendritic cells at priming naive CD4(+) T cells. J Invest Dermatol. 2010;130(5):1345–1354. 20107482 10.1038/jid.2009.424

[50] Mathers AR , Janelsins BM , Rubin JP , Tkacheva OA , Shufesky WJ , Watkins SC , et al. Differential capability of human cutaneous dendritic cell subsets to initiate Th17 responses. J Immunol. 2009;182(2):921–933. 19124735 10.4049/jimmunol.182.2.921

[51] Ouchi T , Kubo A , Yokouchi M , Adachi T , Kobayashi T , Kitashima DY , et al. Langerhans cell antigen capture through tight junctions confers preemptive immunity in experimental staphylococcal scalded skin syndrome. J Exp Med. 2011;208(13):2607–2613. 22143886 10.1084/jem.20111718 PMC3244045

[52] de Witte L , Nabatov A , Pion M , Fluitsma D , de Jong MA , de Gruijl T , et al. Langerin is a natural barrier to HIV‐1 transmission by Langerhans cells. Nat Med. 2007;13(3):367–371. 17334373 10.1038/nm1541

[53] GeurtsvanKessel CH , Willart MA , van Rijt LS , Muskens F , Kool M , Baas C , et al. Clearance of influenza virus from the lung depends on migratory langerin+ CD11b‐ but not plasmacytoid dendritic cells. J Exp Med. 2008;205(7):1621–1634. 18591406 10.1084/jem.20071365 PMC2442640

[54] Wong E , Montoya B , Stotesbury C , Ferez M , Xu RH , Sigal LJ . Langerhans cells orchestrate the protective antiviral innate immune response in the lymph node. Cell Rep. 2019;29(10):3047–3059.e3. 31801072 10.1016/j.celrep.2019.10.118 PMC6927544

[55] Brewig N , Kissenpfennig A , Malissen B , Veit A , Bickert T , Fleischer B , et al. Priming of CD8+ and CD4+ T cells in experimental leishmaniasis is initiated by different dendritic cell subtypes. J Immunol. 2009;182(2):774–783. 19124720 10.4049/jimmunol.182.2.774

[56] Bobr A , Olvera‐Gomez I , Igyarto BZ , Haley KM , Hogquist KA , Kaplan DH . Acute ablation of Langerhans cells enhances skin immune responses. J Immunol. 2010;185(8):4724–4728. 20855870 10.4049/jimmunol.1001802 PMC3050031

[57] Gomez de Aguero M , Vocanson M , Hacini‐Rachinel F , Taillardet M , Sparwasser T , Kissenpfennig A , et al. Langerhans cells protect from allergic contact dermatitis in mice by tolerizing CD8(+) T cells and activating Foxp3(+) regulatory T cells. J Clin Invest. 2012;122(5):1700–1711. 22523067 10.1172/JCI59725 PMC3336977

[58] Kitashima DY , Kobayashi T , Woodring T , Idouchi K , Doebel T , Voisin B , et al. Langerhans cells prevent autoimmunity via expansion of keratinocyte antigen‐specific regulatory T cells. EBioMedicine. 2018;27:293–303. 29307572 10.1016/j.ebiom.2017.12.022 PMC5828466

[59] Lu Q , Lemke G . Homeostatic regulation of the immune system by receptor tyrosine kinases of the tyro 3 family. Science. 2001;293(5528):306–311. 11452127 10.1126/science.1061663

[60] Bauer T , Zagorska A , Jurkin J , Yasmin N , Koffel R , Richter S , et al. Identification of Axl as a downstream effector of TGF‐beta1 during Langerhans cell differentiation and epidermal homeostasis. J Exp Med. 2012;209(11):2033–2047. 23071254 10.1084/jem.20120493 PMC3478937

[61] King JK , Philips RL , Eriksson AU , Kim PJ , Halder RC , Lee DJ , et al. Langerhans cells maintain local tissue tolerance in a model of systemic autoimmune disease. J Immunol. 2015;195(2):464–476. 26071559 10.4049/jimmunol.1402735 PMC4562401

[62] van der Aar AM , Picavet DI , Muller FJ , de Boer L , van Capel TM , Zaat SA , et al. Langerhans cells favor skin flora tolerance through limited presentation of bacterial antigens and induction of regulatory T cells. J Invest Dermatol. 2013;133(5):1240–1249. 23389393 10.1038/jid.2012.500

[63] Bieber T . Atopic dermatitis: an expanding therapeutic pipeline for a complex disease. Nat Rev Drug Discov. 2022;21(1):21–40. 34417579 10.1038/s41573-021-00266-6 PMC8377708

[64] Ye C , Gu H , Li M , Chen R , Xiao X , Zou Y . Air pollution and weather conditions are associated with daily outpatient visits of atopic dermatitis in Shanghai, China. Dermatology. 2022;238:1–11. 35313304 10.1159/000522491

[65] Silverberg NB . Typical and atypical clinical appearance of atopic dermatitis. Clin Dermatol. 2017;35(4):354–359. 28709565 10.1016/j.clindermatol.2017.03.007

[66] Bieber T . Atopic dermatitis. N Engl J Med. 2008;358:1483–1494. 18385500 10.1056/NEJMra074081

[67] Bieber T . How to define atopic dermatitis? Dermatol Clin. 2017;35(3):275–281. 28577796 10.1016/j.det.2017.02.001

[68] de Lusignan S , Alexander H , Broderick C , Dennis J , McGovern A , Feeney C , et al. Atopic dermatitis and risk of autoimmune conditions: population‐based cohort study. J Allergy Clin Immunol. 2022;150:709–713. 35469843 10.1016/j.jaci.2022.03.030

[69] Hülpüsch C , Weins AB , Traidl‐Hoffmann C , Reiger M . A new era of atopic eczema research: advances and highlights. Allergy. 2021;76(11):3408–3421. 34407212 10.1111/all.15058

[70] Wollenberg A , Kinberger M , Arents B , Aszodi N , Avila Valle G , Barbarot S , et al. European guideline (EuroGuiDerm) on atopic eczema – part II: non‐systemic treatments and treatment recommendations for special AE patient populations. J Eur Acad Dermatol Venereol. 2022;36(11):1904–1926. 36056736 10.1111/jdv.18429

[71] Bataille V , Lens M , Spector TD . The use of the twin model to investigate the genetics and epigenetics of skin diseases with genomic, transcriptomic and methylation data. J Eur Acad Dermatol Venereol. 2012;26(9):1067–1073. 22243446 10.1111/j.1468-3083.2011.04444.x

[72] Paternoster L , Standl M , Waage J , Baurecht H , Hotze M , Strachan DP , et al. Multi‐ancestry genome‐wide association study of 21,000 cases and 95,000 controls identifies new risk loci for atopic dermatitis. Nat Genet. 2015;47(12):1449–1456. 26482879 10.1038/ng.3424 PMC4753676

[73] Zheng C , Shi Y , Zou Y . T cell co‐stimulatory and co‐inhibitory pathways in atopic dermatitis. Front Immunol. 2023;14:1081999. 36993982 10.3389/fimmu.2023.1081999 PMC10040887

[74] Czarnowicki T , He H , Krueger JG , Guttman‐Yassky E . Atopic dermatitis endotypes and implications for targeted therapeutics. J Allergy Clin Immunol. 2019;143(1):1–11. 30612663 10.1016/j.jaci.2018.10.032

[75] Koh LF , Ong RY , Common JE . Skin microbiome of atopic dermatitis. Allergol Int. 2022;71(1):31–39. 34838450 10.1016/j.alit.2021.11.001

[76] Peng W , Benfadal S , Yu C , Wenzel J , Oldenburg J , Novak N . JAK1/2 inhibitor but not IL‐4 receptor alpha antibody suppresses allergen‐mediated activation of human basophils in vitro. Allergy. 2022;77:2253–2256. 35460281 10.1111/all.15322

[77] Peng W , Kwiek B , Yu C , Garbi N , Allam JP , Oldenburg J , et al. Infiltration and clustering of major histocompatibility complex II(+) antigen‐presenting cells in the skin of patients with atopic dermatitis. J Invest Dermatol. 2020;141:939–942. 32898509 10.1016/j.jid.2020.07.032

[78] Bieber T . The pro‐ and anti‐inflammatory properties of human antigen‐presenting cells expressing the high affinity receptor for IgE (Fc epsilon RI). Immunobiology. 2007;212(6):499–503. 17544834 10.1016/j.imbio.2007.03.001

[79] Bieber T . Allergen‐specific sublingual immunotherapy: less mystic, more scientific. Allergy. 2006;61(2):149–150. 16409189 10.1111/j.1398-9995.2006.01042.x

[80] Werner Y , Lindberg M . Transepidermal water loss in dry and clinically normal skin in patients with atopic dermatitis. Acta Derm Venereol. 1985;65(2):102–105. 2408409

[81] De Benedetto A , Rafaels NM , McGirt LY , Ivanov AI , Georas SN , Cheadle C , et al. Tight junction defects in patients with atopic dermatitis. J Allergy Clin Immunol. 2011;127(3):773–786.e7. 21163515 10.1016/j.jaci.2010.10.018 PMC3049863

[82] Palmer CN , Irvine AD , Terron‐Kwiatkowski A , Zhao Y , Liao H , Lee SP , et al. Common loss‐of‐function variants of the epidermal barrier protein filaggrin are a major predisposing factor for atopic dermatitis. Nat Genet. 2006;38(4):441–446. 16550169 10.1038/ng1767

[83] Leyden J , Marples R , Kligman A . Staphylococcus aureus in the lesions of atopic dermatitis. Br J Dermatol. 1974;90:525–530. 4601016 10.1111/j.1365-2133.1974.tb06447.x

[84] Simpson EL , Villarreal M , Jepson B , Rafaels N , David G , Hanifin J , et al. Patients with atopic dermatitis colonized with *Staphylococcus aureus* have a distinct phenotype and endotype. J Invest Dermatol. 2018;138(10):2224–2233. 29604251 10.1016/j.jid.2018.03.1517 PMC6153055

[85] Kong HH , Oh J , Deming C , Conlan S , Grice EA , Beatson MA , et al. Temporal shifts in the skin microbiome associated with disease flares and treatment in children with atopic dermatitis. Genome Res. 2012;22(5):850–859. 22310478 10.1101/gr.131029.111 PMC3337431

[86] Khadka VD , Key FM , Romo‐Gonzalez C , Martinez‐Gayosso A , Campos‐Cabrera BL , Geronimo‐Gallegos A , et al. The skin microbiome of patients with atopic dermatitis normalizes gradually during treatment. Front Cell Infect Microbiol. 2021;11:720674. 34631601 10.3389/fcimb.2021.720674 PMC8498027

[87] Liu X , Qin Y , Dong L , Han Z , Liu T , Tang Y , et al. Living symbiotic bacteria‐involved skin dressing to combat indigenous pathogens for microbiome‐based biotherapy toward atopic dermatitis. Bioact Mater. 2023;21:253–266. 36157249 10.1016/j.bioactmat.2022.08.019 PMC9477860

[88] Myles IA , Williams KW , Reckhow JD , Jammeh ML , Pincus NB , Sastalla I , et al. Transplantation of human skin microbiota in models of atopic dermatitis. JCI Insight. 2016;1(10). 10.1172/jci.insight.86955 PMC4963067 27478874

[89] Huang X , Li F , Wang F . Neural regulation of innate immunity in inflammatory skin diseases. Pharmaceuticals (Basel). 2023;16(2). 10.3390/ph16020246 PMC9961653 37259392

[90] Tobin D , Nabarro G , Baart de la Faille H , van Vloten WA , van der Putte SC , Schuurman HJ . Increased number of immunoreactive nerve fibers in atopic dermatitis. J Allergy Clin Immunol. 1992;90(4 Pt 1):613–622. 1383306 10.1016/0091-6749(92)90134-n

[91] Fujii M , Akita K , Mizutani N , Nabe T , Kohno S . Development of numerous nerve fibers in the epidermis of hairless mice with atopic dermatitis‐like pruritic skin inflammation. J Pharmacol Sci. 2007;104(3):243–251. 17609584 10.1254/jphs.fp0070436

[92] Bigliardi‐Qi M , Lipp B , Sumanovski LT , Buechner SA , Bigliardi PL . Changes of epidermal mu‐opiate receptor expression and nerve endings in chronic atopic dermatitis. Dermatology. 2005;210(2):91–99. 15724090 10.1159/000082563

[93] Groneberg DA , Serowka F , Peckenschneider N , Artuc M , Grutzkau A , Fischer A , et al. Gene expression and regulation of nerve growth factor in atopic dermatitis mast cells and the human mast cell line‐1. J Neuroimmunol. 2005;161(1–2):87–92. 15748947 10.1016/j.jneuroim.2004.12.019

[94] Takano N , Sakurai T , Ohashi Y , Kurachi M . Effects of high‐affinity nerve growth factor receptor inhibitors on symptoms in the NC/Nga mouse atopic dermatitis model. Br J Dermatol. 2007;156(2):241–246. 17223862 10.1111/j.1365-2133.2006.07636.x

[95] Schuller E , Teichmann B , Haberstok J , Moderer M , Bieber T , Wollenberg A . In situ expression of the costimulatory molecules CD80 and CD86 on langerhans cells and inflammatory dendritic epidermal cells (IDEC) in atopic dermatitis. Arch Dermatol Res. 2001;293(9):448–454. 11758787 10.1007/s004030100263

[96] Elentner A , Finke D , Schmuth M , Chappaz S , Ebner S , Malissen B , et al. Langerhans cells are critical in the development of atopic dermatitis‐like inflammation and symptoms in mice. J Cell Mol Med. 2009;13(8B):2658–2672. 19538461 10.1111/j.1582-4934.2009.00797.x PMC8183941

[97] Xiao C , Zhu Z , Zhang C , Gao J , Luo Y , Fang H , et al. A population of dermal Langerin(+) dendritic cells promote the inflammation in mouse model of atopic dermatitis. Front Immunol. 2022;13:981819. 36304463 10.3389/fimmu.2022.981819 PMC9592551

[98] Cabanillas B . Dupilumab for atopic dermatitis‐from clinical trials to molecular and cellular mechanisms. Dermatitis. 2023;34(1):21–28. 36705657 10.1089/DERM.0000000000000905

[99] Ebner S , Nguyen VA , Forstner M , Wang YH , Wolfram D , Liu YJ , et al. Thymic stromal lymphopoietin converts human epidermal Langerhans cells into antigen‐presenting cells that induce proallergic T cells. J Allergy Clin Immunol. 2007;119(4):982–990. 17320941 10.1016/j.jaci.2007.01.003

[100] Soumelis V , Reche PA , Kanzler H , Yuan W , Edward G , Homey B , et al. Human epithelial cells trigger dendritic cell mediated allergic inflammation by producing TSLP. Nat Immunol. 2002;3(7):673–680. 12055625 10.1038/ni805

[101] Jarrett R , Salio M , Lloyd‐Lavery A , Subramaniam S , Bourgeois E , Archer C , et al. Filaggrin inhibits generation of CD1a neolipid antigens by house dust mite‐derived phospholipase. Sci Transl Med. 2016;8(325):325ra18. 10.1126/scitranslmed.aad6833 PMC4872823 26865566

[102] Furue M , Nindl M , Kawabe K , Nakamura K , Ishibashi Y , Sagawa K . Epitopes for CD1a, CD1b, and CD1c antigens are differentially mapped on Langerhans cells, dermal dendritic cells, keratinocytes, and basement membrane zone in human skin. J Am Acad Dermatol. 1992;27(3):419–426. 1383294 10.1016/0190-9622(92)70211-w

[103] Nakajima S , Igyarto BZ , Honda T , Egawa G , Otsuka A , Hara‐Chikuma M , et al. Langerhans cells are critical in epicutaneous sensitization with protein antigen via thymic stromal lymphopoietin receptor signaling. J Allergy Clin Immunol. 2012;129(4):1048–1055.e6. 22385635 10.1016/j.jaci.2012.01.063 PMC4600611

[104] Ogawa M , Berger PA , McIntyre OR , Clendenning WE , Ishizaka K . IgE in atopic dermatitis. Arch Dermatol. 1971;103(6):575–580. 4104056

[105] Pellefigues C . IgE autoreactivity in atopic dermatitis: paving the road for autoimmune diseases? Antibodies (Basel). 2020;9(3). 10.3390/antib9030047 PMC7551081 32911788

[106] Bieber T , de la Salle H , Wollenberg A , Hakimi J , Chizzonite R , Ring J , et al. Human epidermal Langerhans cells express the high affinity receptor for immunoglobulin E (FceRI). J Exp Med. 1992;175:1285–1290. 1533242 10.1084/jem.175.5.1285 PMC2119213

[107] Bieber T , Ring J . In vivo modulation of the high affinity receptor for IgE (FceRI) on human epidermal Langerhans cells. Int Arch Allergy Immunol. 1992;99:204–207. 34167195 10.1159/000236249

[108] Wollenberg A , Kraft S , Hanau D , Bieber T . Immunomorphological and ultrastructural characterization of Langerhans cells and a novel, inflammatory dendritic epidermal cell (IDEC) population in lesional skin of atopic eczema. J Invest Dermatol. 1996;106(3):446–453. 8648175 10.1111/1523-1747.ep12343596

[109] Reich K , Heine A , Hugo S , Blaschke V , Middel P , Kaser A , et al. Engagement of the fc epsilon RI stimulates the production of IL‐16 in Langerhans cell‐like dendritic cells. J Immunol. 2001;167(11):6321–6329. 11714796 10.4049/jimmunol.167.11.6321

[110] Novak N , Valenta R , Bohle B , Laffer S , Haberstok J , Kraft S , et al. FcepsilonRI engagement of Langerhans cell‐like dendritic cells and inflammatory dendritic epidermal cell‐like dendritic cells induces chemotactic signals and different T‐cell phenotypes in vitro. J Allergy Clin Immunol. 2004;113(5):949–957. 15131579 10.1016/j.jaci.2004.02.005

[111] Simon D , Braathen LR , Simon HU . Eosinophils and atopic dermatitis. Allergy. 2004;59(6):561–570. 15147438 10.1111/j.1398-9995.2004.00476.x

[112] Matsui K , Wirotesangthong M , Nishikawa A . Percutaneous application of peptidoglycan from *Staphylococcus aureus* induces eosinophil infiltration in mouse skin. Clin Exp Allergy. 2007;37(4):615–622. 17430360 10.1111/j.1365-2222.2007.02673.x

[113] Anderton H , Chopin M , Dawson CA , Nutt SL , Whitehead L , Silke N , et al. Langerhans cells are an essential cellular intermediary in chronic dermatitis. Cell Rep. 2022;39(10):110922. 35675765 10.1016/j.celrep.2022.110922

[114] Proksch E , Brasch J , Sterry W . Integrity of the permeability barrier regulates epidermal Langerhans cell density. Br J Dermatol. 1996;134(4):630–638. 8733362

[115] Yoshida K , Kubo A , Fujita H , Yokouchi M , Ishii K , Kawasaki H , et al. Distinct behavior of human Langerhans cells and inflammatory dendritic epidermal cells at tight junctions in patients with atopic dermatitis. J Allergy Clin Immunol. 2014;134(4):856–864. 25282566 10.1016/j.jaci.2014.08.001

[116] Yoshida T , Beck LA , De Benedetto A . Skin barrier defects in atopic dermatitis: from old idea to new opportunity. Allergol Int. 2022;71(1):3–13. 34916117 10.1016/j.alit.2021.11.006 PMC8934597

[117] Klufa J , Bauer T , Hanson B , Herbold C , Starkl P , Lichtenberger B , et al. Hair eruption initiates and commensal skin microbiota aggravate adverse events of anti‐EGFR therapy. Sci Transl Med. 2019;11(522). 10.1126/scitranslmed.aax2693 31826981

[118] Lee HJ , Kim TG , Kim SH , Park JY , Lee M , Lee JW , et al. Epidermal barrier function is impaired in Langerhans cell‐depleted mice. J Invest Dermatol. 2019;139(5):1182–1185. 30448384 10.1016/j.jid.2018.10.036

[119] Herman KE , Yoshida T , Hughson A , Grier A , Gill SR , Beck LA , et al. IL‐17‐dependent dysregulated cutaneous immune homeostasis in the absence of the Wiskott‐Aldrich syndrome protein. Front Immunol. 2022;13:817427. 35265075 10.3389/fimmu.2022.817427 PMC8900519

[120] Luo Y , Wang S , Liu X , Wen H , Li W , Yao X . Langerhans cells mediate the skin‐induced tolerance to ovalbumin via Langerin in a murine model. Allergy. 2019;74(9):1738–1747. 30964950 10.1111/all.13813

[121] Hrestak D , Matijasic M , Cipcic Paljetak H , Ledic Drvar D , Ljubojevic Hadzavdic S , Peric M . Skin microbiota in atopic dermatitis. Int J Mol Sci. 2022;23(7). 10.3390/ijms23073503 PMC8998607 35408862

[122] Chen H , Zhao Q , Zhong Q , Duan C , Krutmann J , Wang J , et al. Skin microbiome, metabolome and skin phenome, from the perspectives of skin as an ecosystem. Phenomics. 2022;2(6):363–382. 36939800 10.1007/s43657-022-00073-y PMC9712873

[123] Meisel JS , Sfyroera G , Bartow‐McKenney C , Gimblet C , Bugayev J , Horwinski J , et al. Commensal microbiota modulate gene expression in the skin. Microbiome. 2018;6(1):20. 29378633 10.1186/s40168-018-0404-9 PMC5789709

[124] Shen W , Li W , Hixon JA , Bouladoux N , Belkaid Y , Dzutzev A , et al. Adaptive immunity to murine skin commensals. Proc Natl Acad Sci USA. 2014;111(29):E2977–E2986. 25002505 10.1073/pnas.1401820111 PMC4115524

[125] Scholz F , Badgley BD , Sadowsky MJ , Kaplan DH . Immune mediated shaping of microflora community composition depends on barrier site. PLoS One. 2014;9(1):e84019. 24416190 10.1371/journal.pone.0084019 PMC3885526

[126] Ubags ND , Trompette A , Pernot J , Nibbering B , Wong NC , Pattaroni C , et al. Microbiome‐induced antigen‐presenting cell recruitment coordinates skin and lung allergic inflammation. J Allergy Clin Immunol. 2021;147(3):1049–1062.e7. 32679208 10.1016/j.jaci.2020.06.030

[127] Seneschal J , Clark RA , Gehad A , Baecher‐Allan CM , Kupper TS . Human epidermal Langerhans cells maintain immune homeostasis in skin by activating skin resident regulatory T cells. Immunity. 2012;36(5):873–884. 22560445 10.1016/j.immuni.2012.03.018 PMC3716276

[128] Liu X , Zhang X , Zhang J , Luo Y , Xu B , Ling S , et al. Activation of aryl hydrocarbon receptor in Langerhans cells by a microbial metabolite of tryptophan negatively regulates skin inflammation. J Dermatol Sci. 2020;100(3):192–200. 33082071 10.1016/j.jdermsci.2020.10.004

[129] Iwamoto K , Numm TJ , Koch S , Herrmann N , Leib N , Bieber T . Langerhans and inflammatory dendritic epidermal cells in atopic dermatitis are tolerized toward TLR2 activation. Allergy. 2018;73(11):2205–2213. 29672867 10.1111/all.13460

[130] Kröger S , Neuber K , Gruseck E , Ring J , Abeck D . Pityrosporum ovale extracts increase interleukin‐4, interleukin‐10 and IgE synthesis in patients with atopic eczema. Acta Derm Venereol. 1995;75(5):357–360. 8615051 10.2340/0001555575357360

[131] Selander C , Zargari A , Mollby R , Rasool O , Scheynius A . Higher pH level, corresponding to that on the skin of patients with atopic eczema, stimulates the release of Malassezia sympodialis allergens. Allergy. 2006;61(8):1002–1008. 16867055 10.1111/j.1398-9995.2006.01108.x

[132] Radi G , Campanti A , Diotallevi F , Martina E , Marani A , Offidani A . A systematic review of atopic dermatitis: the intriguing journey starting from physiopathology to treatment, from laboratory bench to bedside. Biomedicine. 2022;10(11). 10.3390/biomedicines10112700 PMC9688004 36359220

[133] Misery L , Chesnais M , Merhand S , Aubert R , Bru MF , Legrand C , et al. Perceived stress in four inflammatory skin diseases: an analysis of data taken from 7273 adult subjects with acne, atopic dermatitis, psoriasis or hidradenitis suppurativa. J Eur Acad Dermatol Venereol. 2022;36(8):e623–e626. 35181931 10.1111/jdv.18016 PMC9546193

[134] Keller JJ . Cutaneous neuropeptides: the missing link between psychological stress and chronic inflammatory skin disease? Arch Dermatol Res. 2023;315:1875–1881. 36700961 10.1007/s00403-023-02542-4

[135] Lee HJ , Lee GN , Lee JH , Han JH , Han K , Park YM . Psychological stress in parents of children with atopic dermatitis: a cross‐sectional study from the Korea National Health and nutrition examination survey. Acta Derm Venereol. 2023;103:adv00844. 36621921 10.2340/actadv.v103.2242 PMC9885276

[136] Lugovic‐Mihic L , Mestrovic‐Stefekov J , Cvitanovic H , Bulat V , Duvancic T , Pondeljak N , et al. The COVID‐19 pandemic and recent earthquake in Zagreb together significantly increased the disease severity of patients with atopic dermatitis. Dermatology. 2023;239(1):91–98. 36049473 10.1159/000525901 PMC9892999

[137] Shi Y‐Y , Wei Q , Ma X , Zhang Y , Wang L , Shi H‐J . Maternal affective and stress‐related factors during pregnancy affect the occurrence of childhood allergic diseases: a Shanghai MCPC study. J Psychosom Res. 2023;165:111142. 36630818 10.1016/j.jpsychores.2022.111142

[138] Doss ALN , Smith PG . Langerhans cells regulate cutaneous innervation density and mechanical sensitivity in mouse footpad. Neurosci Lett. 2014;578:55–60. 24970748 10.1016/j.neulet.2014.06.036 PMC4119831

[139] Zhang S , Edwards TN , Chaudhri VK , Wu J , Cohen JA , Hirai T , et al. Nonpeptidergic neurons suppress mast cells via glutamate to maintain skin homeostasis. Cell. 2021;184(8):2151–2166.e16. 33765440 10.1016/j.cell.2021.03.002 PMC8052305

[140] Albertin G , Ravara B , Kern H , Zampieri S , Loefler S , Hofer C , et al. Trauma of peripheral innervation impairs content of epidermal Langerhans cells. Diagnostics (Basel). 2022;12(3). 10.3390/diagnostics12030567 PMC8947052 35328120

[141] Duchesne M , Danigo A , Richard L , Vallat J‐M , Attarian S , Gonnaud P‐M , et al. Skin biopsy findings in patients with CMT1A: baseline data from the CLN‐PXT3003‐01 study provide new insights into the pathophysiology of the disorder. J Neuropathol Exp Neurol. 2018;77(4):274–281. 29408953 10.1093/jnen/nly001

[142] Beresford L , Orange O , Bell EB , Miyan JA . Nerve fibres are required to evoke a contact sensitivity response in mice. Immunology. 2004;111(1):118–125. 14678206 10.1111/j.1365-2567.2003.01786.x PMC1782395

[143] Cevikbas F , Lerner EA . Physiology and pathophysiology of itch. Physiol Rev. 2020;100(3):945–982. 31869278 10.1152/physrev.00017.2019 PMC7474262

[144] Katsuno M , Aihara M , Kojima M , Osuna H , Hosoi J , Nakamura M , et al. Neuropeptides concentrations in the skin of a murine (NC/Nga mice) model of atopic dermatitis. J Dermatol Sci. 2003;33(1):55–65. 14527739 10.1016/s0923-1811(03)00155-5

[145] Salomon J , Baran E . The role of selected neuropeptides in pathogenesis of atopic dermatitis. J Eur Acad Dermatol Venereol. 2008;22(2):223–228. 18211417 10.1111/j.1468-3083.2007.02399.x

[146] Hosoi J , Murphy GF , Egan CL , Lerner EA , Grabbe S , Asahina A , et al. Regulation of Langerhans cell function by nerves containing calcitonin gene‐related peptide. Nature. 1993;363(6425):159–163. 8483499 10.1038/363159a0

[147] Torii H , Hosoi J , Asahina A , Granstein RD . Calcitonin gene‐related peptide and Langerhans cell function. J Investig Dermatol Symp Proc. 1997;2(1):82–86. 10.1038/jidsymp.1997.16 9487021

[148] Staniek V , Misery L , Peguet‐Navarro J , Abello J , Doutremepuich JD , Claudy A , et al. Binding and in vitro modulation of human epidermal Langerhans cell functions by substance P. Arch Dermatol Res. 1997;289(5):285–291. 9164639 10.1007/s004030050194

[149] Mathers AR , Tckacheva OA , Janelsins BM , Shufesky WJ , Morelli AE , Larregina AT . In vivo signaling through the neurokinin 1 receptor favors transgene expression by Langerhans cells and promotes the generation of Th1‐ and Tc1‐biased immune responses. J Immunol. 2007;178(11):7006–7017. 17513750 10.4049/jimmunol.178.11.7006

[150] Toyoda M , Nakamura M , Makino T , Hino T , Kagoura M , Morohashi M . Nerve growth factor and substance P are useful plasma markers of disease activity in atopic dermatitis. Br J Dermatol. 2002;147(1):71–79. 12100187 10.1046/j.1365-2133.2002.04803.x

